# 

**Published:** 1881-06

**Authors:** 


### Table of Contents,

ORIGINAL COMMUNICATIONS

Carbolic Acid in Cancer. By J. G. Westmoreland, M.D., Atlanta, Georgia.......... 129

REPORTS OF SOCIETIES.

Boston Society for Medical Improvement.......... 133
Medical Society of the County of New York.......... 136
Kentucky State Medical Society.......... 140
Eighty-third Annual Meeting of the Medical and Chirurgical Faculty of Maryland.......... 146
Baltimore Academy of Medicine.......... 154

SELECTIONS.

One Phase of the Germ Theory.......... 161
The Value of Local Treatment in Syphilitic Ulceration of the Larynx and Pharynx.......... 165
A Question of Ethics.......... 172
Indigestion Due to Nervous Influences.......... 174

EDITORIAL.

Public Charities.......... 183
The Physiological Antagonism of Remedies.......... 185

BIBLIOGRAPHICAL.

Hernia: Strangulated and Reducible, with Cure by Subcutaneous Injections.......... 186
A Medico-Legal Treatise on Malpractice, Medical Evidence and Insanity.......... 187
The Diseases of Children.......... 187

EXCERPTA.

The Accuracy of Clinical Thermometers.......... 188
Eucalyptus Globulus. Its Value in Chronic Diseases of the Stomach and Bowels.......... 189
Doctors' Mistakes and Blunders.......... 190

